# Influence of Maitake (*Grifola frondosa*) Particle Sizes on Human Mesenchymal Stem Cells and *In Vivo* Evaluation of Their Therapeutic Potential

**DOI:** 10.1155/2020/8193971

**Published:** 2020-03-06

**Authors:** Dinesh K. Patel, Yu-Ri Seo, Sayan Deb Dutta, Ok Hwan Lee, Ki-Taek Lim

**Affiliations:** ^1^Department of Biosystems Engineering, College of Agriculture and Life Science, The Institute of Forest Science, Kangwon National University, Chuncheon-24341, Republic of Korea; ^2^Department of Food Science and Biotechnology, Kangwon National University, Chuncheon-24341, Republic of Korea

## Abstract

Maitake (*Grifola frondosa*) mushroom has received an enormous amount of attention as a dietary supplement due to its high nutritional values. The particle sizes of *G. frondosa* mushrooms were monitored by a classifying mill. *β*-Glucans are the bioactive component of the mushroom, and it was revealed through Fourier transform infrared spectroscopy (FTIR), proton and carbon nuclear magnetic resonance (^1^H and ^13^C-NMR), matrix-assisted laser desorption/ionization, and time-of-flight (MALDI-TOF) spectrometry. The biocompatibility of *G. frondosa* particles, as well as induced osteogenesis of hMSCs, was evaluated through WST-1 assay and alizarin staining (ARS) technique, respectively. Notably, enhanced cell viability was noted in the presence of *G. frondosa*. Significantly improved calcium deposition has observed from hMSCs with *G. frondosa,* suggesting to their mineralization potential. The expression of osteogenic related gene markers was examined in the presence of *G. frondosa* through real-time polymerase chain reaction (qPCR) technique. The upregulation of osteogenic gene markers in the presence of *G. frondosa* particles was indicating their superior osteogenic potential. Besides, *G. frondosa* also activated the secretion of various kinds of proteins from the hMSCs indicating their potential for tissue engineering applications. Enhanced secretion of different immunoglobulins was observed in rat serum in the presence of *G. frondosa*, further demonstrating their therapeutic nature. Therefore, *G. frondosa* is effective for enhanced osteogenesis and can be utilized as a natural, edible, and osteogenic agent.

## 1. Introduction

Mushroom, a member of the Fungi kingdom, has drawn a considerable amount of interest for biomedical applications due to its anti-inflammatory, antimicrobial, antidiabetic, cardiovascular-protective, hepatoprotective, and anticancer properties. It is well-known that mushrooms have the efficiency in regulating the immune system as well as macrophages, T cells, dendritic cells (DC), natural killer (NK) cells, and hematopoietic stem cell activities through several ways by activating the phagocytic activity, generation of reactive oxygen species, inflammatory mediators, and cytokines production [1–4]. Cytokines play critical roles in the regulation of homeostasis of the individual through cell differentiation, proliferation, apoptosis, inflammatory reactions, as well as immune responses [5]. The maitake mushroom (*Grifola frondosa*) is a popular edible mushroom in Japan and also known as hen-of-the-woods, sheep's head, and ram's head [6]. Mushrooms are the rich source of proteins, fibers, as well as vitamins, and it is not easy to distinguish between edible and medicinal mushrooms because many edible mushrooms have therapeutic properties and many medicinal mushrooms are also edible [7–9]. Chitin and *β*-glucans are the two essential constituents of the mushroom's cell wall; and out of this, *β*-glucans (1 ⟶ 3), *β* (1 ⟶ 4), and *β* (1 ⟶ 6) make mushrooms a vital material to use as therapeutic agents [10–12]. Besides, mushrooms also have polysaccharides, polysaccharide-protein complexes, polyphenols, terpenoids, agaritine, ergosterol, and selenium in their structure [13, 14]. Glucans are heterogeneous polysaccharides, where large numbers of glucose units are linked together differently. *β*-Glucans derived from various sources have varying degrees of branching, branch linkage, and backbone linkage in their structure that affects the biological activity. Hence, it is necessary to identify and characterize *β*-glucans with better therapeutic and immune-stimulatory activities [15]. It was observed that glucans stimulated defense reactions against infections and cancer [16, 17]. Besides this, reduction in stress and cholesterol level, hypoglycemic effects, and improvements of ulcerative colitis were also observed in the presence of the glucans [18–21]. Moreover, the production of tumor necrosis factor-*α* (TNF-*α*), interleukin-6 (IL-6, IL-1), and interferon-*γ* (IFN-*γ*) known as pro-inflammatory cytokines were also observed in the presence of *β*-glucans by activating the macrophage cells [22, 23].

The study aimed to evaluate the effects of different particle sizes of *G. frondosa* on human mesenchymal stem cells (hMSCs) in terms of cell viability, mineralization, osteogenesis, and immunoglobulin secretion. The *β*-glucan was the chief constituent of the ethanol extract of the *G. frondosa* and was characterized through FTIR, ^1^H-NMR, ^13^C-NMR, and MALDI-TOF spectrometry. No adverse effects were exhibited by *G. frondosa* particles towards the hMSCs, indicating their biocompatibility. The activity of hMSCs can be easily tuned by considering a suitable particle size of *G. frondosa* for various applications.

## 2. Materials and Methods

### 2.1. Preparation of the Ultrafine Ground Materials

The ultrafine ground mushroom powders (*Grifola frondosa*, Republic of Korea) were obtained by air classifying mill (ACM-AL, Daega, Republic of Korea). The mushroom samples were pulverized by an impeller rotating at high speed in the air classifying milling machine. In the process, the feed mass was kept constant at 13 kg, and the rotating speed was set at 3600 g. The ultrafine ground mushrooms were analyzed through a classifier analysis software (Mastersizer S v3.10, Malvern, UK). Three particle size of the mushroom powder was fractionated (10-20, 20-30, and 30-40 *μ*m, respectively).

### 2.2. Extraction and Structural Characterization of *β*-Glucan

The extraction of *β*-glucan from *G*. *frondosa* was done as described earlier in somewhere else from another edible mushroom [24]. In brief, *G*. *frondosa* particles were dried in a hot air oven at 60°C for 48 h followed by the aqueous (4% NaOH solution) treatment with continuous mechanical stirring for 1 h at 90°C. After this, polysaccharide was precipitated with ethanol solution at room temperature and incubated it for 24 h at 4°C. The precipitate was separated through centrifugation (4000 rpm, 30 min), and the supernatant was removed. After this, the precipitate was collected and washed with ethanol and acetone several times. The obtained materials were dialyzed with cellulose bag (12000-14000 Da, Cellu Sep, Texas, USA) to remove the small soluble molecules for 3 days, followed by centrifugation and freeze-dried. The yield of the material was ~30% (w/w). The structural properties of the extracted material were elucidated by FTIR (Perkin-Elmer, Buckinghamshire, UK), in the scanning range of 500-4000 cm^−1^ at a resolution of 4 cm^−1^, ^1^H-NMR measurement (600 MHz, FT-NMR, Bruker) in Me_2_SO-D_2_O (6 : 1, 15 mg/mL) at 70°C, matrix-assisted laser deposition/ionization (time-of-flight), and MALDI-TOF mass spectrometry (Bruker Autoflex speed TOF/TOF) as described earlier [25]. The extract was used only for the chemical characterization of the *β*-glucan present in *G*. *frondosa*, and the other study was performed with *G*. *frondosa* particles.

### 2.3. Cell Viability

Cell viability experiment was performed to evaluate the cytotoxicity of different sizes of mushroom powders. The human mesenchymal stem cells (hMSCs) were received from the bone (Korean Cell Line Bank, Republic of Korea) and cultured with proliferative medium (90% Dulbecco's modified Eagle medium (DMEM), 10% fetal bovine serum, and 1% antibiotics). The hMSCs were incubated at 37°C in a humidified atmosphere of 5% CO_2_ for desired periods. Cell viability was examined through the WST-1 assay (EZ-Cytox Cell Viability Assay Kit, Daeillab Service Co., Ltd, Republic of Korea). For this, different particle sizes of *G*. *frondosa* (10, 20, 50, and 100 *μ*g/mL) samples were incubated with hMSCs in 96-well plates for 24 h. After this, the samples were treated with EZ-Cytox and further incubated for 4 h to form the soluble formazan. The concentration of formed formazan was measured by a spectrophotometer (Infinite® M Nano 200 Pro; TECAN, Switzerland) with an absorbance value of 450 nm (625 nm as a reference value). All the samples were performed in triplicate fashion, and the data are given at average ODs ± standard deviations. The cell medium without the sample was set as a control.

### 2.4. Alkaline Phosphatase (ALP) Activity

The hMSCs (5 × 10^3^) were cultured in DMEM media containing 50 *μ*g/mL ascorbic acid, 10 mM *β*-glycerophosphate, and 100 nM dexamethasone (Sigma-Aldrich, USA) for 7 days to evaluate the ALP activity. Briefly, the cells were fixed in 10% formalin solution (Duksan Chemicals Co., Gyeonggi-do, Republic of Korea), followed by rinsing with 1× PBS for two times. These cells were incubated with 0.1% Triton X-100 for 5 min, followed by staining with Leukocyte Alkaline Phosphatase Kit (Sigma-Aldrich, USA) according to the manufacturer's protocol. The ALP activity in cell lysates was determined by reacting with para-Nitrophenylphosphate (p-NPP) as a substrate in an assay buffer containing 5 mM MgCl_2_ and Na_2_CO_3_. The absorbance was taken by using a spectrophotometer with an absorbance value of 405 nm.

### 2.5. RNA Preparation and Real-Time PCR Analysis

The RNA preparation and real-time PCR was performed as earlier reported by our group [26]. For this, 1.0 × 10^6^ cells were incubated in a 6 mm culture dish for 7 and 14 days under differentiation media. The RNeasy Mini Kit (Qiagen, Valencia, CA, USA) was utilized to prepare the RNA as per manufacturer's instruction to synthesis the cDNA through reverse transcriptase (Superscript II Preamplification System, Invitrogen, Gaithersburg, MD, USA). The real-time PCR was accomplished with SYBR Green PCR Master Mix (ABI Prism 7500 sequence detection system; Applied Biosystems, Warrington, UK) with the reaction conditions 40 cycles for 15 s of denaturation at 95°C and 1 min of amplification at 60°C. All experiments were implemented in triplicate fashion and normalized it with housekeeping gene hypoxanthine-guanine phosphoribosyltransferase (*HPRT*). The relative RNA expression levels in hMSCs from control and *G. frondosa* treated samples were compared in a histogram. The expression levels of *ALP*, *BSP*, *OPN*, *OCN*, *COL1*, *OSX*, and *RUNX2* were evaluated. The specific primer sets used for this analysis are given in Supplementary [Supplementary-material supplementary-material-1].

### 2.6. Antibody Arrays for Growth Factor

The RayBio™ human cytokine array C1 (AAH-CYT-1-2) was purchased from Ray Biotech Inc. (Norcross, GA, USA) for the analysis of cytokine expression. An array membrane could detect 44 different growth factors. The hMSCs with different particle sizes of mushroom powders were seeded into a 100 mm culture plate and incubated for 7 and 14 days. The cell medium without samples was considered as the control. The cells were then starved to exclude the influence of serum cells for 24 h. The cell-free supernatants were obtained and analyzed by the human cytokine arrays. The experiment was conducted according to the manufacturer's instructions. Briefly, the membranes were blocked with blocking buffer and then treated with a sample at 4°C overnight. The membranes were then washed with wash buffer followed by the addition of a biotinylated antibody cocktail. The membranes were incubated at room temperature for 2 h and accomplished with washing. The obtained samples were incubated with the HRP-Streptavidin at room temperature for 2 h. Finally, the membranes were rewashed and developed by incubation with detection buffer for 5 min. The chemiluminescence of the membranes was measured using the Chemidoc XRS system (BR170-8265, Bio-Rad, USA). Relative protein expression was obtained by comparing the signal intensities.

### 2.7. *In Vivo* Study

The rats (ICR; male, 248.78-249.67 g, six weeks old) were received from the Orient Bio Inc. (Seongnam, Republic of Korea). These animals were kept in a well-insulated room at an ambient temperature of 21 ± 2°C for experimental periods with an automatically controlled 12 h light/12 h dark cycle (lights off at 20 : 00) at 35-65% of humidity. The experimental animals were divided into four groups, including control. An animal without *G. frondosa* dose was treated as the control. The animals were treated with *G. frondosa* in a ratio of 300 mg/kg. The secretions of the different immunoglobulins such as IgA, IgG, and IgM in the blood serum of rats were measured after the end of the administration. IgA, IgG, and IgM were measured using an ELISA kit, no E-EL-R0516, R0518, and R0519, respectively. During the experimental period, the body weights of the experimental animals have been measured every day. The whole process of experimental animals was approved by the Animal Experimental Ethics Committee of Kangwon National University (Institutional Animal Care and Use Committee of Kangwon National University No. KW-170922-1).

### 2.8. Statistical Analysis

All statistical analyses carried out using SPSS Statistics (IBM SPSS Statistics 23, IBM Inc., USA). Statistical significance between control and treatment groups was compared with a one-way analysis of variance (ANOVA). Statistical significance was considered ^∗^*p* < 0.05.

## 3. Results and Discussion

### 3.1. Structural Analysis of *β*-Glucan

**Figure 1:**
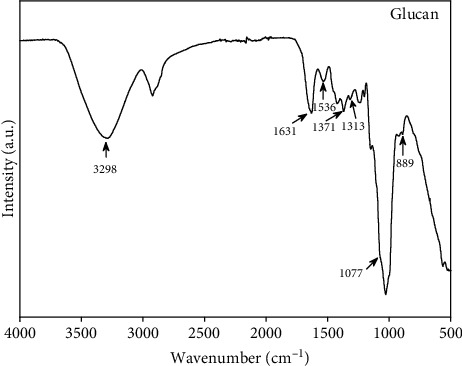
The FTIR spectrum of *β*-glucan extracted from *G. frondosa.*

FTIR spectroscopy is an important analytical tool that is widely utilized in the structural evaluation of polysaccharides [27]. FTIR spectroscopy enables useful information related to the position and anomeric configuration of glycosidic bonds in glucans. It has been noted that the fruiting structures of mushrooms primarily contain branched (1 ⟶ 3) (1 ⟶ 6)-*β*-D-glucan and linear (1 ⟶ 3)-*α*-D-glucan in their structure [28]. The “sugar region” (1200-950 cm^−1^) and “anomeric region” (950-750 cm^−1^) are the important features for the structural elucidation of polysaccharides. The FTIR spectrum of the *β*-glucan extracted from *G. frondosa* is given in (Figure 1). The appearance of the absorption peak at 3298 cm^−1^ in the FTIR spectrum indicates the presence of hydroxyl (-OH) functional groups in the structure. The presence of the FTIR absorption peaks at near 1371, 1313, 1077, and 889 cm^−1^ clearly demonstrates the characteristics of *β*-glucan in the structure [29]. The FTIR absorption peaks at 1631 and 1536 cm^−1^ were due to the presence of the amide (I) and amide (II) moiety in the extracted material. The absorption peak at 889 cm^−1^ is a characteristic peak of (1 ⟶ 3)-*β*-D-glucan. The FTIR result clearly shows that the extracted material has mainly consisted of *β*-D-glucan.

**Figure 2:**
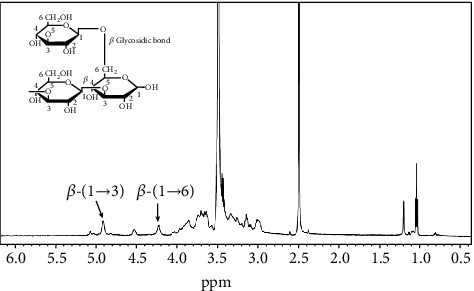
A ^1^H-NMR spectrum of *β*-glucan extracted from *G. frondosa* indicating the presence of *β*-(1 ⟶ 3) and *β*-(1 ⟶ 6)-D-glucan moiety.

**Figure 3:**
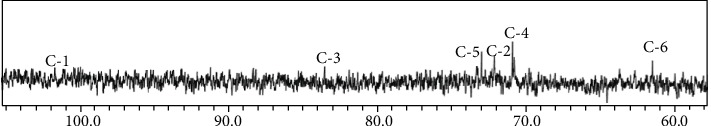
The ^13^C-NMR spectrum of G. frondosa extracted *β-*glucan at room temperature in deuterated Me_2_SO and D_2_O (6 : 1) solvents.

Proton NMR spectrum of *β*-glucan extracted from *G. frondosa* is given in (Figure 2). It is well-established that an anomeric proton signal of glucan has appeared in the range of 4-6 ppm, and this position is highly influenced by the chemical moieties present in their structure [30]. The appearance of the peaks at~ 4.89 and 4.28 ppm in the ^1^H-NMR spectrum indicates the presence of *β*-(1 ⟶ 3) and *β*-(1 ⟶ 6)-D-glucan proton in the extracted material, respectively [28, 31, 32]. Furthermore, the branching signal of *β*-(1 ⟶ 3)-branched *β*-(1 ⟶ 6)-D-glucan is observed at ~4.55 ppm in the proton spectrum [30]. The 400-MHz ^13^C-NMR spectrum of the extracted *β*-glucan from *G. frondosa* in deuterated Me_2_SO and D_2_O (6 : 1) solvents at room temperature is given in (Figure 3). The presence of the anomeric peak at the chemical shift (*δ*) 101.7 ppm clearly indicates the *β*-configuration of glucan. The signal at *δ* 83.5 ppm is assigned to the presence of a C-3 of *β*-(1 ⟶ 3)-D-glucan structure. The presence of the other carbon signals is assigned in the spectrum. The result is good agreement with the previously reported *δ* values of *β-*glucan extracted from the edible mushroom [24].

**Figure 4:**
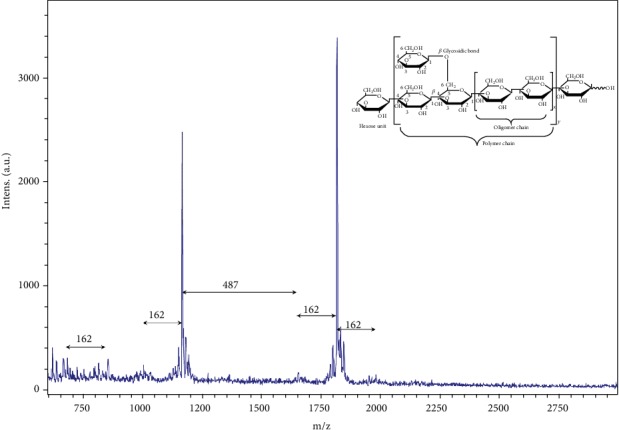
MALDI-TOF mass spectrum of *G. frondosa* glucans using 2, 5-DHB as a matrix.

MALDI-TOF mass spectrum *β*-glucan obtained from *G. frondosa* particles is shown in (Figure 4). MALDI-TOF mass spectrum clearly indicates the presence of the *β*-(1 ⟶ 3) glucan unit in the extract with the molecular mass (between the peaks) gap of approximately 162 Da (one hexose unit) as observations have been reported previously [33, 34]. It has noticed that 2, 5-dihydroxybenzoic acid (2, 5-DHB) matrix facilitates the polysaccharide signals intensity in the MALDI-TOF mass spectrometer. However, it was interesting to note that the molecular gap in-between some peaks were higher than the162 atomic mass unit (a.m.u.), and it was 487 a.m.u. This was three folds higher than one hexose structure, indicating the presence of some oligomer units in the extract [35]. The mass spectrum of *β*-glucan extracted from the *G. frondosa* suggests that glucans are composed of a mixture of glucose units with molecular weights around 679.6~1977. 1 m/z with the degree of polymerization (DP) = 4~12 which is similar to the previously reported values of glucan extracted from mushrooms [36]. Based on these results, we concluded that *β*-glucan has composed of hexose units with *β*-(1 ⟶ 3) and *β*-(1 ⟶ 6)-D-glucan linkage. The presumable structure of glucans is presented inside the mass spectrum.

### 3.2. Particle Size and Cell Viability

**Figure 5:**
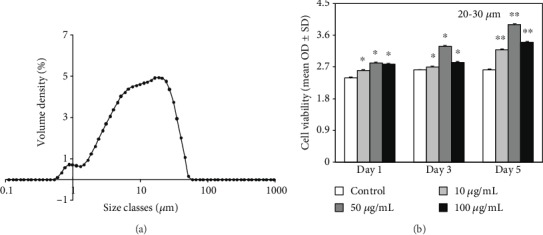
(a) The particle sizes analysis of *G. frondosa* powder air classifying mill and (b) the cell viability of hMSCs in the presence of different concentrations of *G. frondosa* (20-30 *μ*m) at indicated time intervals (^∗^*p* < 0.05 and ^∗∗^*p* < 0.01).

The particle size analysis of *G. frondosa* powder was accomplished through an air classifying mill, and the result is given in (Figure 5(a)). Here, we used the three different sizes (10-20, 20-30, and 30-40 *μ*m) of *G. frondosa* particle for further studies. The granulometric distribution of used *G. frondosa* powder is given in Supplementary [Supplementary-material supplementary-material-1]. It is well-known that cellular behavior is profoundly affected by the particle size, surface charge, as well as coating and attachment of chemical moiety in the particles [37]. The cell viability data of hMSCs in the presence of different concentrations (10, 50, and 100 *μ*g/mL) of 20-30 *μ*m size of *G. frondosa* after different time intervals is given in (Figure 5(b)). Notably, better cell viability was observed in the presence of *G. frondosa* particles after 1 day of treatment indicated the biocompatibility of *G. frondosa* towards the hMSCs. Furthermore, an enhancement in cell viability was observed in *G. frondosa* treated media than the control after 5 days of treatment, indicating their better biocompatibility. Among these concentrations, 50 *μ*g/mL exhibited higher cell viability than the other suggested that the 50 *μ*g/mL concentration is optimum for cellular activity. Moreover, out of these, 20-30 *μ*m size of *G. frondosa* favored more cell viability after 5 days of incubation than other sizes. The cell viability results for the other sizes of *G. frondosa* (10-20 and 30-40 *μ*m) at different concentrations and time intervals are shown in Supplementary [Supplementary-material supplementary-material-1]. No adverse effects were noted on hMSCs viability in the presence of other sizes of *G. frondosa*, showing their biocompatibility. However, a slight decrease in cell viability was noted in these samples than the 20-30 *μ*m size of *G. frondosa.* This is attributed to the presence of a higher amount of *β*-glucan in 20-30 *μ*m size of *G. frondosa* particles that facilitate the better growth of cells [35].

### 3.3. Mineralization and Real-Time PCR Analysis

**Figure 6:**
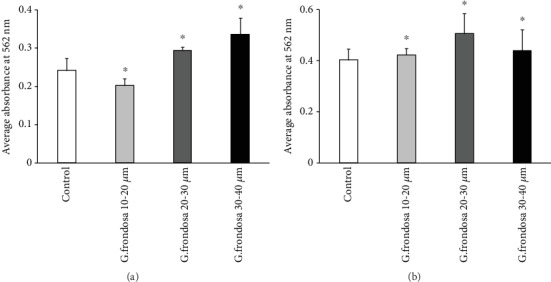
Evaluation of calcium deposition by alizarin staining technique from hMSCs in the presence of *G. frondosa* particles after (a) 7 days and (b) 14 days of incubation (^∗^*p* < 0.05).

**Figure 7:**
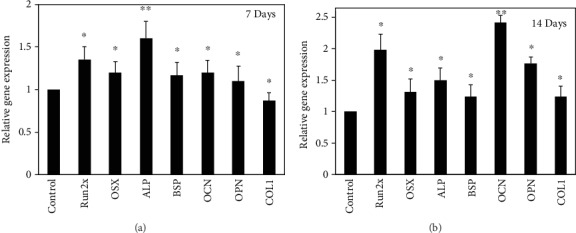
Relative gene expression from hMSCs in the presence of *G. frondosa* (20-30 *μ*m) (a) after 7 and (b) 14 days of treatment (^∗^*p* < 0.05 and ^∗∗^*p* < 0.01).

It is well-known that the activity of hMSCs is profoundly affected by the surrounding environments, and further, it can be optimized by utilizing the appropriate conditions. Stem cells are considered the most critical cells in the field of tissue engineering due to their differentiation potential. Stem cells can be differentiated into various other cells, including osteoblasts, chondrocytes, and adipocytes [38, 39]. Osteogenic differentiation of hMSCs can be monitored by alkaline phosphatase activity (APA), calcium deposition, and cell number [40]. Calcium deposition in the surrounding medium by hMSCs was evaluated through the alizarin staining technique and given in (Figures 6(a) and 6(b)). Significantly, enhanced calcium deposition was observed in the presence of *G. frondosa* after 7 days of incubation, and this further improved after 14 days of incubation, suggesting to their potential for osteogenic differentiation of hMSCs. Interestingly, 20-30 *μ*m size *G. frondosa* particles exhibited higher calcium deposition compared to other sizes after 14 days of incubation due to the presence of higher content of bioactive *β*-glucan in 20-30 *μ*m size *G. frondosa* that induced the greater osteogenesis of hMSCs [35]. The qualitative analysis of ALP activity in the presence of 20-30 *μ*m size of *G. frondosa* at different concentrations after 7 days of treatment are given in Supplementary [Supplementary-material supplementary-material-1]. The medium without the material was considered as a control. The higher ALP activity was noted in 50 *μ*g/mL treated media than the other as well as the control, indicating their superior osteogenic potential. Bone formation is a widely complicated biological process and directly related to the expression of various osteogenic associated gene markers [41]. We have examined the expression levels of *RUNX2*, *OSX*, *ALP*, *BSP*, *OCN*, *OPN*, and *COL1* in the presence of 50 *μ*g/mL concentration of 20-30 *μ*m size *G. frondosa* particles to confirm the osteogenesis process at the molecular level after 7 and 14 days of treatment and their expression values are given in (Figures 7(a) and 7(b)). We have taken the 50 *μ*g/mL concentrations of *G. frondosa* in this study because, at this concentration, cell viability was high than other concentrations. The relative osteogenic gene expression value for control was one in this experiment. Collagen1 (*COL1*) is the most prominent protein that occurred in the bone matrix and generated during the proliferation of osteoblast cells [42]. The expression of the *COL1* gene marker from hMSCs shows the formation of bone cells in the presence of *G. frondosa.* This expression potential was higher after 14 days of treatment compared to the control demonstrating their better osteogenic efficiency. The ALP is another important gene marker that indicates the occurrence of preosteoblasts and osteoblast cells during the differentiation of the cells [43]. The ALP activity was high in *G. frondosa* treated medium than the control suggested their superior osteogenic ability. The higher expression of runt-related transcription x2 (*RUNX2*) gene marker from the sample treated medium than the control indicating the osteogenic differentiation ability of *G. frondosa*. *RUNX2* is an early osteogenic marker, and it has been observed that the osteogenesis has not occurred without the expression of *RUNX2.* It controls the osteogenic differentiation of the cells and also termed as Cbfal or AML3 transcription factor [44]. It has been noted that the expression and activity of RUNX2 marker are profoundly affected by other transcription factors and protein-protein of protein-nucleic acid interactions. The bone resorption process is facilitated in the excess amount of *RUNX2* factor [45]. Bone sialoprotein (BSP) is a class of “small integrin-binding ligand N-linked glycoprotein” (SIBLING) protein which occurred in bone and dentin. They play a very crucial role in bone generation, healing, remodeling, and mineralization [46]. The upregulation of the BSP marker in the presence of *G. frondosa* vis-à-vis control after 7 and 14 days of treatment further confirms their osteogenic ability. The expression of OPN and OSX was also higher in *G. frondosa* treated media than the control. OCN is another most significant gene marker for osteogenic differentiation of stem cells. Its maximum expression is occurred during the mineralization and accumulates mineralized bones. OCN expression was high in sample treated condition compared to the control, which further upregulated after 14 days of treatment clearly demonstrates the superior osteogenic potential of *G. frondosa*. These results (mineralization and PCR data) confirm the osteogenic ability of *G. frondosa*, and it can be used as an osteogenic agent in tissue engineering.

### 3.4. Growth Factors

**Figure 8:**
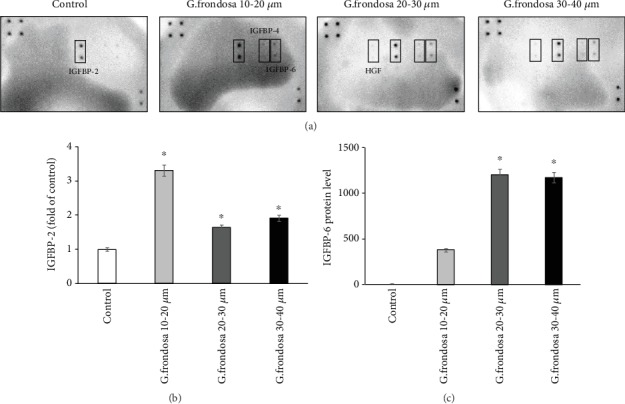
The representative membrane of growth factor from hMSCs activated by *G. frondosa*. (a) Secretion of antibodies in the cell-free supernatants with or without *G. frondosa* treated and quantification of antibody array results for (b) IGFBP-2 and (c) IGFBP-6 (^∗^*p* < 0.05).

**Figure 9:**
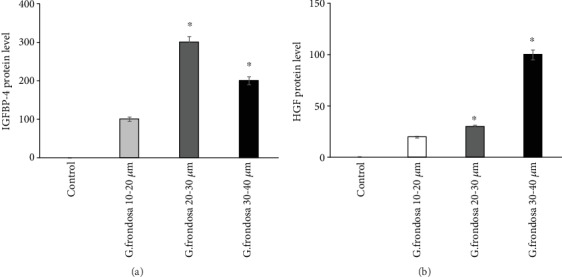
Secretion of (a) IGFBP4 and (b) HGF by hMSCs in the presence of different *G. frondosa* particles (^∗^*p* < 0.05).

Insulin-like growth factors (IGF) with its different binding proteins play vital roles in cell growth, survival, as well as in differentiation, depending on the cell culture microenvironments [47]. A template showing the location of antibodies for protein spotted onto the human growth factor array is given in Supplementary [Supplementary-material supplementary-material-1]. Secretion of various kinds of insulin-like growth factors binding protein (IGFBP) occurred by hMSCs in the presence of *G. frondosa*. IGFs are the small polypeptides that can control the survival, self-renewal, and differentiation potential of stem cells [48]. The secretion of antibodies in the cell-free supernatants with or without *G. frondosa* on the surface of the membrane is shown in (Figure 8(a)). More significant and intensified spots were observed on the membrane surface in the presence of *G. frondosa* than those of control. A comparative study for the secretion of IGFBP2 and IGFBP6 through hMSCs in the presence of different *G. frondosa* particles is shown in (Figures 8(b) and 8(c)). It was observed that a higher amount of IGFBP2 and IGFBP6 secretion occurred in the presence of *G. frondosa.* This was interesting to note that a higher amount of IGFBP2 and IGFBP6 was secreted by hMSCs on 10-20 *μ*m and 20-30 *μ*m size of *G. frondosa,* respectively. Moreover, an increased amount of IGFBP4 secretion was also observed on 20-30 *μ*m size of *G. frondosa*. The secretion of IGFBP4 and HGF is given in (Figures 9(a) and 9(b)). It has been noted that the IGF-1 facilitated the mTOR signaling pathway in order to activate the expression of various osteogenic gene markers, including *RUNX2*, *OCN*, *OSX*, and *COL1* [49]. Our study demonstrated that during the differentiation upregulation of IGFBP 2, 4, and 6 were occurred, which trigger the expression of different osteogenic gene markers. Growth factors also play a vital role in the osteogenesis of stem cells through a different signaling pathway. It has been seen that vascular endothelial growth factor (VEGF) has a direct influence on cell migration and osteogenic differentiation. An enhancement in cell proliferation and differentiation was noted in the presence of VEGF [50]. The cells treated with *G. frondosa* exhibited an increase in calcium deposition, ALP activity, and expression of osteogenic gene markers such as *RUNX2*, *OSX*, *ALP*, *BSP*, *OCN*, *OPN*, and *COL1* than the control. These finding suggested that *G. frondosa* have the potential to express the various kinds of growth factors from hMSCs and consequently causing the osteogenic differentiation of cells by upregulating the osteogenic markers.

### 3.5. *In Vivo* Study

**Figure 10:**
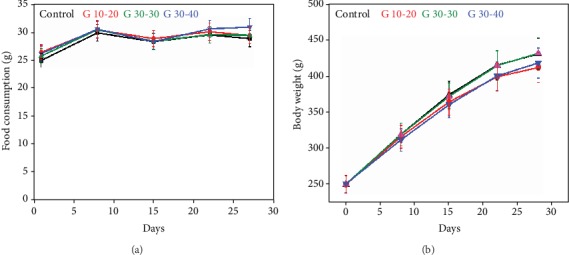
Effect of *G. frondosa* on rat. (a) The food consumption rate of rat in the presence of different *G. frondosa* particles and (b) the change in body weight after consumption of *G. frondosa.*

**Figure 11:**
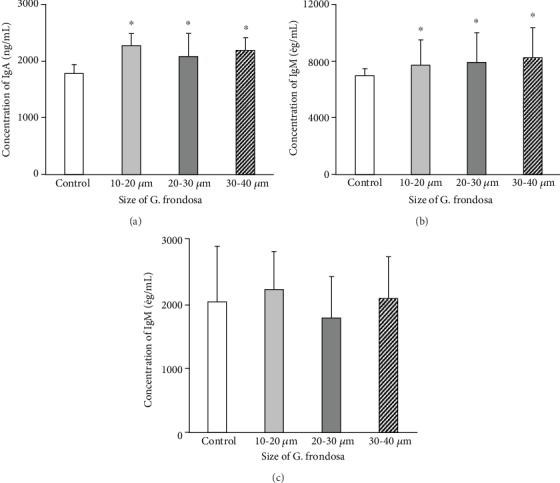
Effect of *G. frondosa* on the secretion of (a) IgA, (b) IgG, and (c) IgM antibodies (^∗^*p* < 0.05).

Food consumption graph of experimental rat in the presence of *G. frondosa* particles and without particles has been shown in (Figure 10(a)). The graph indicates that the food consumption rate was high in the presence of *G. frondosa* particles than control suggesting their biocompatibility. A group summary of food consumption by a rat with standard deviation for entire experimental periods is presented in Table 1. No clinical sign has been observed from a rat during the entire experimental periods, and their daily observation is summarized in Table 2. It was interesting to note that rapid gain in weight occurred in the rat during the experimental periods. However, a decrease in body weight was observed in the presence of *G. frondosa* than control, and this decrease was more effective in small-sized (10-20 *μ*m) particles indicating that weight increase was inhibited by *G. frondosa* as reported earlier [51]. The change in body weight of rat treated in the presence of *G. frondosa* particles is shown in (Figure 10(b)). This decrease in the body weight of rats in the presence of *G. frondosa* is related to their antihypertensive and antidiabetic effects [52]. The effect of *G. frondosa* particles on rat body weight is summarized in Table 3. *G. frondosa* induced secretion of immunoglobulin (Ig) A, G, and M in rat serum is shown in (Figure 11). It is important to note that in all cases, the secretion of the aforementioned Ig was higher than control, further suggesting their therapeutic nature. It is well-known that IgA acts as an anti-inflammatory antibody that regulates the homeostasis in the mucosa [53]. The *G. frondosa* induced secretion of IgA antibody is given in (Figure 11(a)). The secretion of the IgA antibody in the presence of *G. frondosa* was ~1.13 folds higher than control, indicating their improved immunomodulating potential for immune protection. It was noted that the high-affinity IgA antibody protects intestinal mucosal surface through microorganisms, whereas low-affinity IgA antibody plays a crucial role in intestinal lumen against bacteria [54]. The secretion of IgG antibody in the presence of *G. frondosa* is shown in (Figure 11(b)). It was ~1.12 folds higher than control. IgG antibody plays a significant role in the immune response directly or by activating the other immune cells [55]. Therefore, the higher secretion of IgG in serum in the presence of *G. frondosa* further indicating their induced immunomodulating nature that helps to maintain the homeostasis in the body. *G. frondosa* induced production of IgM antibody in rat serum is shown in (Figure 11(c)). The IgM is another antibody that expresses during the early stage of the immune response. It is well-established that *β*-glucan is the active component for the immune-enhancing effect in mushroom, and it has a higher concentration in 20-30 *μ*m size of *G. frondosa* [35]. However, small-sized particles are more effective in penetrating the biological barrier than large-sized and rapidly stabilized in blood circulation [56]. Therefore, in all cases, 10-20 *μ*m size of *G. frondosa* exhibited more immunomodulatory potential than others. Furthermore, these results indicated that *G. frondosa* mushroom has different physiological activities.

## 4. Conclusions

Mushrooms have considered a significant amount of interest from the scientific community due to their high nutritional and medicinal values. It is often to use as a food supplement that induced the immunity of people through their different bioactive components. Notably, improved cell viability was observed in the presence of *G. frondosa* than the control, and this potential was widely affected by the sizes of *G. frondosa* particles. Furthermore, enhanced calcium deposition was noted in the presence of *G. frondosa* particles than those of control, indicating their greater mineralization efficiency. The higher expression of osteogenic gene markers further confirms the superior osteogenic efficacy of *G. frondosa*. Enhanced secretion of various insulin-like growth factor binding proteins (IGFBP) such as IGFBP-2, 4, and 6 demonstrated the better cellular activities of *G. frondosa*. Besides, *G. frondosa* induced secretion of different immunoglobulin in rat serum further suggested their therapeutic nature. It was important to note that this secretion is profoundly affected by the size of mushroom particles. Therefore, the proper size of mushrooms should be used for a better therapeutic result. Future research is needed to examine the relationship between the structures and functions of *G. frondosa* polysaccharides. This study will facilitate the researchers to develop more health-promoting pharmaceuticals and functional food supplements.

## Figures and Tables

**Table 1:** Food consumption in SD rat treated with test articles for experimental period. (Group summary).

Groups	Result	Food consumption (*g*)				
		Day 1	Days 8	Days 15	Days 22	Days 27
Control	Mean	25.02	29.93	28.37	29.58	28.91
	SD	1.84	1.66	1.64	1.98	1.41
	*N*	9	9	9	9	9

10-20 *μ*m (300 mg/kg)	Mean	26.49	30.45	28.93	30.13	29.46
	SD	2.32	0.77	1.53	0.80	0.70
	*N*	9	9	9	9	9

20-30 *μ*m 300 mg/kg)	Mean	25.80	30.48	28.45	29.61	29.49
	SD	1.17	2.11	1.44	2.34	0.78
	*N*	9	9	9	9	9

30-40 *μ*m 300 mg/kg)	Mean	26.25	30.59	28.36	30.64	30.95
	SD	2.29	1.68	1.74	2.67	2.30
	*N*	9	9	9	9	9

**Table 2:** Daily observation in SD rat treated with test articles for the experimental period (group summary).

Groups	*N*	Clinical observation	Days																											
			1	2	3	4	5	6	7	8	9	10	11	12	13	14	15	16	17	18	19	20	21	22	23	24	25	26	27	28
Control	9	No clinical sign	9	9	9	9	9	9	9	9	9	9	9	9	9	9	9	9	9	9	9	9	9	9	9	9	9	9	9	9
10-20 *μ*m (300 mg/kg)	9	No clinical sign	9	9	9	9	9	9	9	9	9	9	9	9	9	9	9	9	9	9	9	9	9	9	9	9	9	9	9	9
20-30 *μ*m (300 mg/kg)	9	No clinical sign	9	9	9	9	9	9	9	9	9	9	9	9	9	9	9	9	9	9	9	9	9	9	9	9	9	9	9	9
30-40 *μ*m (300 mg/kg)	9	No clinical sign	9	9	9	9	9	9	9	9	9	9	9	9	9	9	9	9	9	9	9	9	9	9	9	9	9	9	9	9

**Table 3:** Body weight in SD rat treated with test articles for experimental period (group summary).

Groups	Result	Body weight (*g*)					
		Day 0	Days 8	Days 15	Days 22	Days 28	Days 29
Control	Mean	248.78	318.58	373.76	415.01	430.65	417.20
	SD	8.22	13.17	18.05	22.52	23.19	21.61
	*N*	9	9	9	9	9	9

10-20 *μ*m, 300 mg/kg)	Mean	249.02	315.18	363.83	398.71	411.76	395.07
	SD	7.97	7.23	9.92	17.81	22.92	21.97
	*N*	9	9	9	9	9	9

20-30 *μ*m, 300 mg/kg)	Mean	249.51	318.29	371.47	414.38	431.60	413.65
	SD	7.70	15.20	18.04	20.19	21.62	17.05
	*N*	9	9	9	9	9	9

30-40 *μ*m, 300 mg/kg)	Mean	249.67	310.88	359.93	399.98	418.04	401.27
	SD	7.85	14.55	18.23	23.57	24.48	23.40
	*N*	9	9	9	9	9	9

## Data Availability

The data used to support the findings of this study are available from the corresponding author upon request.

## Supplementary Materials

Supplementary Materials Supplementary Figure 1: the cell viability of hMSCs in the presence of different concentrations of G. frondosa; (a) with 10-20 *μ*m sizes and (b) 30-40 *μ*m sizes at indicated time intervals (^∗^*p* < 0.05). Supplementary Figure 2: the qualitative analysis of ALP activity in the presence of 20-30 *μ*m size of *G. frondosa* at different concentrations after 7 days of treatment. Supplementary Table 1: specified primer sequences used for quantitative real-time polymerase chain reaction (qRT-PCR). Supplementary Table 2: a granulometric distribution of *G. frondosa* samples. Supplementary Table 3: a template showing the location of antibodies for protein spotted onto the human growth factor array.
