# Supplementary material for: Influence of Maitake (Grifola frondosa) Particle Sizes on Human Mesenchymal Stem Cells and In Vivo Evaluation of Their Therapeutic Potential
Source: Biomed Res Int. 2020 Mar 6;2020:8193971. doi: 10.1155/2020/8193971 (PMC7091544; doi:10.1155/2020/8193971)
Supplement: Supplementary Materials — Supplementary Figure 1: the cell viability of hMSCs in the presence of different concentrations of G. frondosa; (a) with 10-20 μm sizes and (b) 30-40 μm sizes at indicated time intervals (∗p < 0.05). Supplementary Figure 2: the qualitative analysis of ALP activity in the presence of 20-30 μm size of G. frondosa at different concentrations after 7 days of treatment. Supplementary Table 1: specified primer sequences used for quantitative real-time polymerase chain reaction (qRT-PCR). Supplementary Table 2: a granulometric distribution of G. frondosa samples. Supplementary Table 3: a template showing the location of antibodies for protein spotted onto the human growth factor array. [file 8193971.f1.docx]

**Supplementary Information:**

**Influence of maitake (*Grifola frondosa*) particle sizes on human mesenchymal stem cells and *in vivo* evaluation of their therapeutic potential**

Dinesh K. Patel,^1†^ Yu-Ri Seo,^1†^ Sayan Deb Dutta,^1^ Ok Hwan Lee,^2^ and Ki-Taek Lim^1*^

*^1^Department of Biosystems Engineering, College of Agriculture and Life Science, The Institute of Forest Science, Kangwon National University, Chuncheon-24341, Republic of Korea.*

*^2^Department of Food Science and Biotechnology, Kangwon National University, Chuncheon- 24341, Republic of Korea.*

†: Equal contributions.

***Corresponding Author:** ktlim@kangwon.ac.kr

*
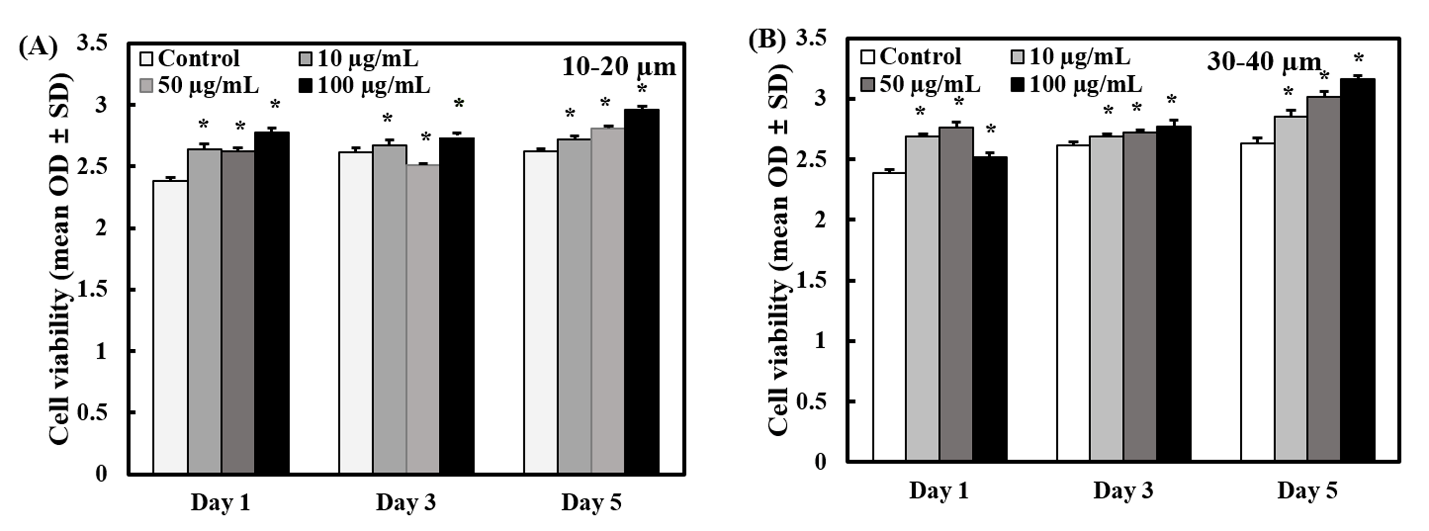
*

**Supplementary Figure 1.**The cell viability of hMSCs in the presence of different concentrations of *G. frondosa*; **(A)** with 10-20 μm sizes, and **(B)** 30-40 μm sizes at indicated time intervals (p^*^<0.05).

*
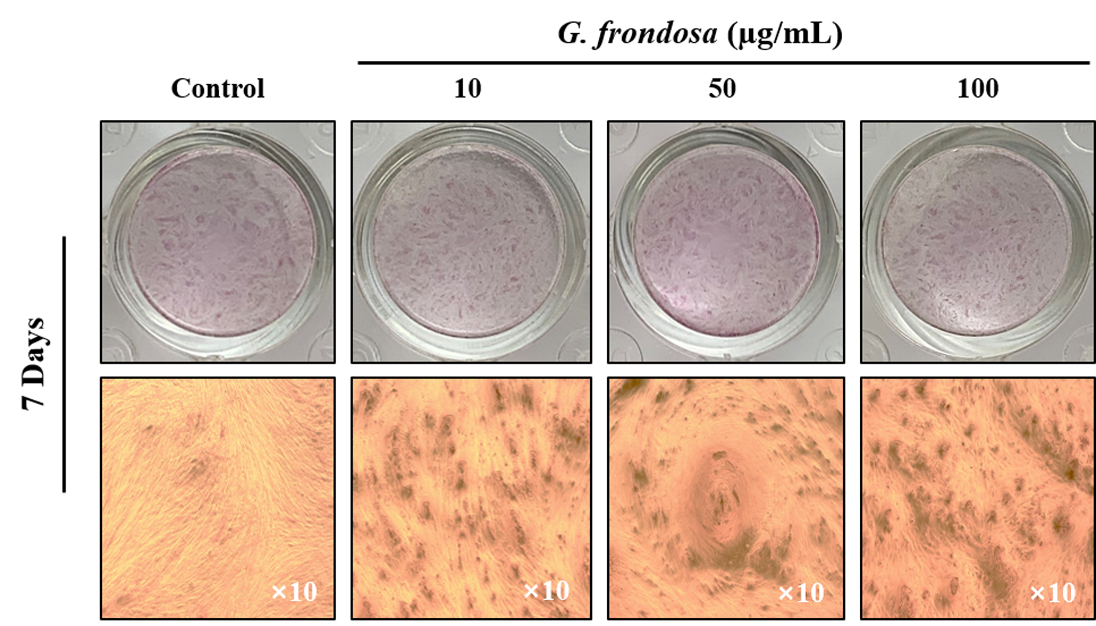
*

**Supplementary Figure 2.** The qualitative analysis of ALP activity in the presence of 20-30 µm size of *G. frondosa* at different concentrations after 7 days of treatment

| **Gene** | **Sequences (5’ to 3’)** |
| --- | --- |
| *β*-actin | 5’-GCGCAAGTACTCTGTGTGGA-3’  5’-ACATCTGCTGGAAGGTGGAC-3’ |
| RUNX2 | 5’-GGACATGCAGTACGAGCTGA-3’  5’-GCAGTGAAGGGCTTCTTGTC-3’ |
| ALP | 5’-GGACATGCAGTACGAGCTGA-3’  5’-GCAGTGAAGGGCTTCTTGTC-3’ |
| OSX | 5’-TGCTTGAGGAGGAAGTTCAC -3’  5’-AGGTCACTGCCCACAGAGTA -3’ |
| BSP | 5’-CAACAGCACAGAGGVAGAAA-3’  5’-CGTACTCCCCCTCGTATTCA-3’ |
| OCN | 5’-GTGCAGAGTCCAGCAAAGGT-3’  5’-TCAGCCAACTCGTCACAGTC-3’ |
| OPN | 5’-CCCACAGACCCTTCCAAGTA-3’  5’-ACACTATCACCTCGGCCATC-3’ |
| COL1 | 5’-CTGACCTTCCTGCGCCTGATGTCC-3’  5’-GTCTGGGGCACCAACGTCCAAGGG-3’ |

**Supplementary Table 1.** Specified primer sequences used for quantitative real-time polymerase chain reaction (qRT-PCR).

**Supplementary Table 2.** A granulometric distribution of *G. frondosa* samples.

**Supplementary Table 3.** A template showing the location of antibodies for protein spotted onto the human growth factor array.
